# The Fungal CYP51s: Their Functions, Structures, Related Drug Resistance, and Inhibitors

**DOI:** 10.3389/fmicb.2019.00691

**Published:** 2019-04-24

**Authors:** Jingxiang Zhang, Liping Li, Quanzhen Lv, Lan Yan, Yan Wang, Yuanying Jiang

**Affiliations:** ^1^Center for New Drug Research, School of Pharmacy, Second Military Medical University, Shanghai, China; ^2^Shanghai Tenth People’s Hospital, Tongji University School of Medicine, Shanghai, China; ^3^Department of Pharmacology, Tongji University School of Medicine, Shanghai, China

**Keywords:** CYP51, antifungal, crystal structure, azoles, resistance

## Abstract

CYP51 (Erg11) belongs to the cytochrome P450 monooxygenase (CYP) superfamily and mediates a crucial step of the synthesis of ergosterol, which is a fungal-specific sterol. It is also the target of azole drugs in clinical practice. In recent years, researches on fungal CYP51 have stepped into a new stage attributing to the discovery of crystal structures of the homologs in *Candida albicans, Cryptococcus neoformans* and *Aspergillus fumigatus*. This review summarizes the functions, structures of fungal CYP51 proteins, and the inhibitors targeting these homologs. In particular, several drug-resistant mechanisms associated with the fungal CYP51s are introduced. The sequences and crystal structures of CYP51 proteins in different fungal species are also compared. These will provide new insights for the advancement of research on antifungal agents.

## Introduction

The incidence of invasive fungal infections has increased during the past three decades, arising more and more concern. For example, statistics show that such incidence ranges from 30 to 40% throughout critical ill patients (Bassetti et al., 2017). Prognosis of invasive fungal infections is serious. Specifically, the mortality rate of systemic *C. albicans* infection is up to 40% (Gunsalus et al., 2016). Risk factors, such as gastrointestinal surgery, widespread use of broad-spectrum antibiotics and popularization of central venous catheters (CVCs) mainly contribute to the invasive *C. albicans* infection. At the same time, non-*Candida* fungal infections should not be neglected, as cryptococcosis is the third prevalent disease in HIV-positive patients, and the mortality of invasive aspergillosis at 30 days in adult ICU patients is estimated to be 33.1% (Lanjewar, 2011; Baddley et al., 2013).

Represented by azole resistance, the continuous emergence of drug-resistant fungal strains has become a serious challenge for public health (Zhang et al., 2017). CYP51 (ERG11) proteins is the target of azoles, which mediates membrane permeability and fluidity by demethylating the 14-α position of lanosterol to form ergosterol (Daum et al., 1998). In attribution to the constant emergence of azole-resistant isolates, a critical understanding of the resistance mechanisms of CYP51 is required for the discovery of novel CYP51 inhibitors.

## Function and Structural Features of Fungal CYP51S

Sterol synthesis is a very ancient pathway. After the appearance of molecular oxygen in the atmosphere, squalene-2,3-epoxide is formed and then cyclized to steroid precursors, such as lanosterol. Under the oxidative removal of methyl groups by CYP51, these precursors were transformed into ergosterol, which is critical in membrane permeability and fluidity in the fungal kingdom (Rohmer et al., 1979; Daum et al., 1998).

Cytochrome P450s (P450s, CYP) are an abundant hemease superfamily. As the first group of enzymes ranked as “superfamily,” cytochrome P450s play an important role in the primary as well as secondary metabolic pathways (Lamb et al., 2007). Until August 2013, this superfamily contained 10 classes, 267 families and over 21,000 members. These members are important for catalyzing the oxidative process of various organic substrates, and play a critical role during heterogeneous metabolism and steroid conversion in biological kingdoms (Hannemann et al., 2007; Munro et al., 2018).

CYP51 proteins belong to the CYP superfamily and is the most conserved protein in it. Unlike other CYP enzymes, CYP51 has a strong specificity. It only catalyzes the demethylation of a very narrow range of substrates, including lanoserol, obtusifoliol, 24,25-dihydrolanosterol, 24-methylenedihydrolanosterol and 4 β-desmethyllanosterol (Lepesheva and Waterman, 2007). The CYP51-involved catalytic reaction consists of three steps, each of which requires one molecule of oxygen and two molecules of NADPH-sourced reduction equivalent. The first two steps are typical cytochrome P450 monooxygenation processes, during which the 14α methyl is converted to methyl alcohol and further converted to methyl aldehyde. And in the last step, the aldehyde group is transformed into formic acid and detached, accompanied with the synthesis of the Δ-14, 15 double bond (Waterman and Lepesheva, 2005).

**FIGURE 1:**
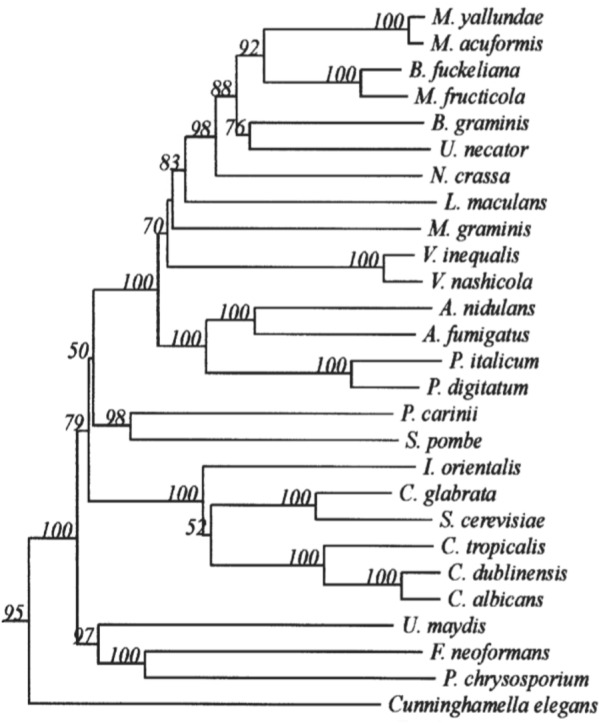
Minor branch of the Fungal CYP51 Phylogenetic Tree. It has been permitted by the copyright holders through RightsLink.

The 14α-demethylase is the only invariant P450 present in all sterol biosynthetic pathways, suggesting that all sterol 14α-demethylases share a common prokaryotic ancestor (Lepesheva and Waterman, 2007). CYP51s are widely distributed in the fungal kingdom. However, in different species of fungi, there are still differences in the types and subtypes, as shown in the phylogenetic tree (Režen et al., 2004) (Figure 1). Only one CYP51 gene exists in the pathogenic fungi *C. albicans*, which belongs to the *Ascomycota Saccharomycotina* (Hawkins et al., 2014). In contrast, 2 or 3 *CYP51* genes are commonly contained in the *Ascomycota Pezizomycotina* genomes, including *CYP51A* and *CYP51B*. *CYP51C* is exclusive in *Fusarium* spp. (Becher et al., 2011). Some *Aspergillus* Spp. such as *A. fumigatus* carries only one CYP51A and one CYP51B protein, while other *Aspergillus* species such as *A. flavus* and *A. terreus* carry a third paralogous gene, which is a copy of *CYP51A* or *CYP51B*. Studies on *Aspergillus fumigatus* have shown that *CYP51B* is constitutively expressed, while *CYP51A* is expressed in an inducible manner. Neither *CYP51A* nor *CYP51B* is essential for *in vitro* growth and virulence, and only the simultaneous inactivation of both genes is lethal (Hu et al., 2007; Hargrove et al., 2015).

A recent study also showed that CYP51 might have a number of indirect functions. In *C. albicans*, the deletion of *CYP51* (*ERG11*) reduces mycelial elongation and invasive growth, and causes defects of reactive oxygen elimination, resulting in reduced virulence *in vivo*. It is worth paying special attention that *ERG11*-deficient fungi are more susceptible to phagocytosis by macrophages, which indicates that CYP51 may be critical for the immune escape process in fungi (Wu et al., 2018).

Present in all animals, plants, fungi, in some protozoa and bacteria, the CYP51 protein located in the inner face of the endoplasmic reticulum is a membrane monospanning enzyme (Noel, 2012). And its N-terminus includes an amphipathic helix, which links the catalytic subunit to the lipid bilayer (Monk et al., 2014). Besides crystallographic structures of CYP51 proteins from some protozoa and bacteria, and the ligand-free CYP51 proteins from Homo sapiens (3JUV) and complexed with ketoconazole (3LD6) deposited in Protein Data Bank (PDB) (Strushkevich et al., 2010). X-ray structures of CYP51 proteins of some fungi have already been described in literature, including CYP51 proteins from *Saccharomyces cerevisiae* complexed with the substrate lanosterol (4LXJ) and complexed with itraconazole (5EQB) (Monk et al., 2014), voriconazole (5HS1) and fluconazole (4WMZ) (Sagatova et al., 2015), from *C. albicans* complexed with posaconazole (5FSA) and the tetrazole-based antifungal drug candidate VT1161 (VT1) (5TZ1) (Hargrove et al., 2017a), and CYP51B structure in complex with the VNI derivative (6CR2) and a tetrazole-based inhibitor VT-1598 (5FRB) from *A. fumigates* (Hargrove et al., 2017b).

**FIGURE 2:**
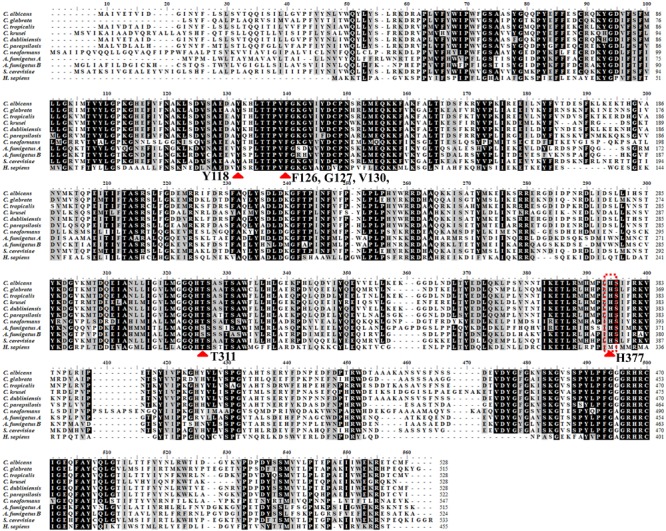
Structure alignment of primary structure of CYP51 from different organisms > 80%. Structure alignment of primary structure of CYP51 from different organisms: *H. sapiens, S. cerevisiae, C. albicans, C. glabrata, C*. *tropicalis, C. krusei, C. dubliniensis, C. parapsilosis, A. fumigatus*, and *Cryptococcus neoformans*. The residues identical in all CYP51s are marked with black, the light gray show the residues conserved in more than 80% sequences. The sequences were from NCBI and CGD database and were aligned using the ClustalW2 program. Red arrow means the most conserved CYP51 active amino acid sites among diffierent species.

Together with information from the analysis of multiple-sequence alignment of CYP51 proteins from human and fungi including *S. cerevisiae, C. albicans, C. glabrata, C. tropicalis, C. krusei, C. dubliniensis, C. parapsilosis, A. fumigatus and Cryptococcus neoformans* showing that the identity varied between 36.5 and 93.9% among them (Table 1). The comprehensive comparative analysis of three-dimensional structures uncovered the basic understanding how CYP51 enzymes might maintain their conservation in human and fungi. As shown in Figure 2, the majority of amino-acids residues conserved in the folding of chains might play an essential structural role for their enzymatic function. The residues forming the surface of CYP51 proteins active site were highly conserved, such as Y118, F126, G127, V130 and T311 from the CYP51 proteins helix B’, B” helical turn and Helix I signature regions, respectively (Lepesheva and Waterman, 2011; Hargrove et al., 2017a; Keniya et al., 2018).

**Table 1:** Comparison of identity (similarity) % of amino acid residue sequence of CYP51 from different organisms calculated using ClustalW2.

	*Homo sapiens*	*Saccharomyces cerevisiae*	*Candida albicans*	*Candida glabrata*	*Candida tropicalis*	*Candida krusei*	*Candida dubliniensis*	*Candida parapsilosis*	*Cryptococcus neoformans*	*Aspergillus fumigatus A*	*Aspergillus fumigatus B*
*H. sapiens*	–	38.0 (56.5)	39.6 (54.7)	37.5 (55.6)	40.0 (54.7)	37.5 (56.8)	37.8 (54.1)	40.3 (58.0)	36.5 (54.8)	38.1 (55.8)	40.1 (58.6)
*S. cerevisiae*	–	–	64.5 (78.1)	84.1 (92.4)	65.5 (77.7)	61.8 (75.4)	63.8 (78.4)	66.6 (80.1)	44.0 (66.1)	49.4 (68.9)	51.1 (71.6)
*C. albicans*	–	–	–	64.5 (77.7)	83.1 (91.5)	61.5 (76.9)	93.9 (98.1)	74.0 (86.0)	46.0 (66.2)	48.3 (66.5)	49.9 (68.0)
*C. glabrata*	–	–	–	–	64.7 (76.1)	62.8 (77.7)	65.0 (77.9)	66.9 (79.1)	43.4 (64.8)	47.6 (66.3)	52.2 (71.0)
*C. tropicalis*	–	–	–	–	–	63.2 (77.2)	81.8 (91.5)	74.6 (85.8)	46.6 (67.5)	48.9 (66.9)	48.6 (66.2)
*C. krusei*	–	–	–	–	–	–	61.4 (77.6)	64.8 (78.8)	45.5 (63.6)	46.9 (66.6)	47.9 (68.2)
*C. dubliniensis*	–	–	–	–	–	–	–	72.5 (85.8)	45.1 (66.2)	48.3 (66.7)	47.7 (65.9)
*C. parapsilosis*	–	–	–	–	–	–	–	–	48.6 (68.5)	49.5 (68.3)	48.3 (67.4)
*C. neoformans*	–	–	–	–	–	–	–	–	–	47.4 (62.6)	48.3 (65.5)
*A. fumigatus A*	–	–	–	–	–	–	–	–	–	–	63.6 (77.4)
*A. fumigatus B*	–	–	–	–	–	–	–	–	–	–	–

*A. fumigatus A, Aspergillus fumigatus CYP51A; *A. fumigatus* B, *Aspergillus fumigatus* CYP51B.*

**FIGURE 3:**
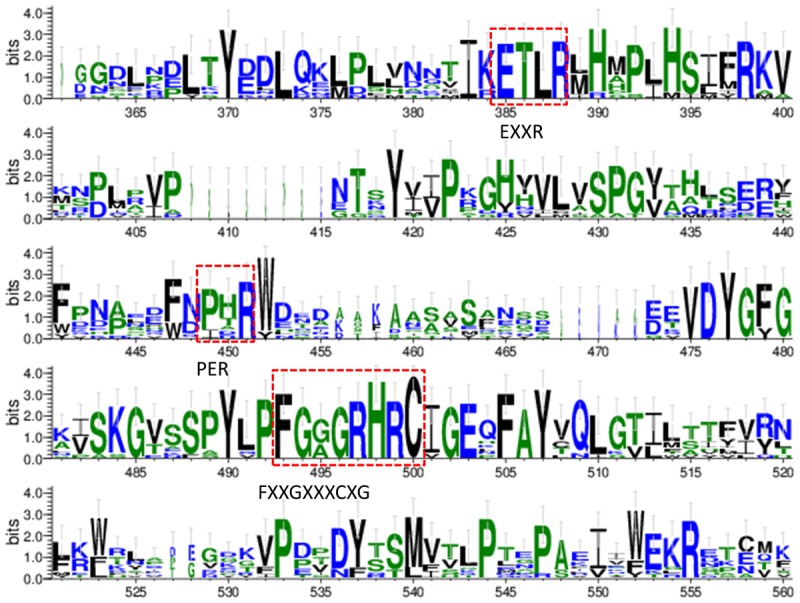
Sequence logos of the conserved CYP motifs from the tested fungi and human’s CYP51. The sequences were from NCBI and CGD database and the consensus logos were generated by WebLogo (http://weblogo.threeplusone.com/create.cgi).

**FIGURE 4:**
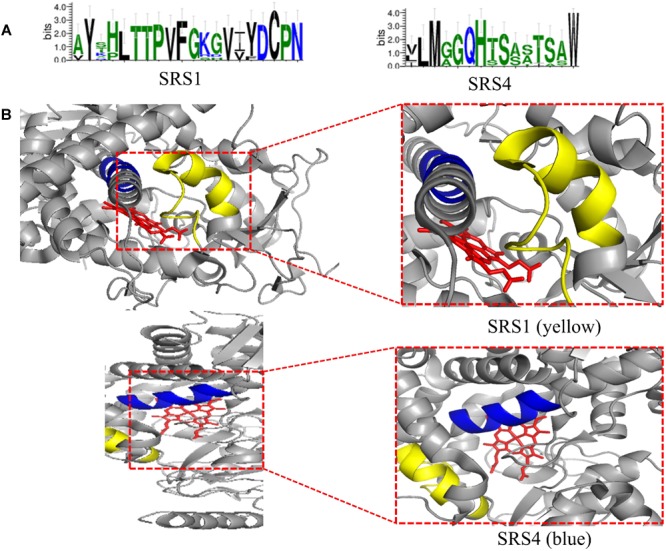
**(A)** Predicted SRS1 (the positions 117–134) and SRS4 (the positions 305–314) of tested fungal CYP51 (*S. cerevisiae, C. albicans, C. glabrata, C. tropicalis, C. krusei, C. dubliniensis, C. parapsilosis, A. fumigatus*, and *Cryptococcus neoformans*). The sequences were from NCBI and CGD database and the consensus logos were generated by WebLogo (http://weblogo.threeplusone.com/create.cgi); **(B)** Location of SRS1(yellow) and SRS4 (blue) in *C. albicans* CYP51 (5v5z). HEME is shown in red. They are obtained from the published crystal data file from the PDB database, and then displayed by PyMOL Version 1.5.0.3.

The conserved amino acid sequences can be classified into three motifs and six putative substrate recognition sites (SRS). Among the three motifs, the most conserved FXXGXXXCXG is a heme binding domain containing a heme axial Cys ligand; and the E–R–R triad, formed by the motifs EXXR and PER, contributes to locking the heme pocket into position and to guarantee stabilization of the core structure (Figure 3). And among the six putative SRSs, the most thoroughly studied SRS1 and SRS4 can be used as landmarks of the fungal CYP51 (Lepesheva and Waterman, 2007) (Figure 4).

**FIGURE 5:**
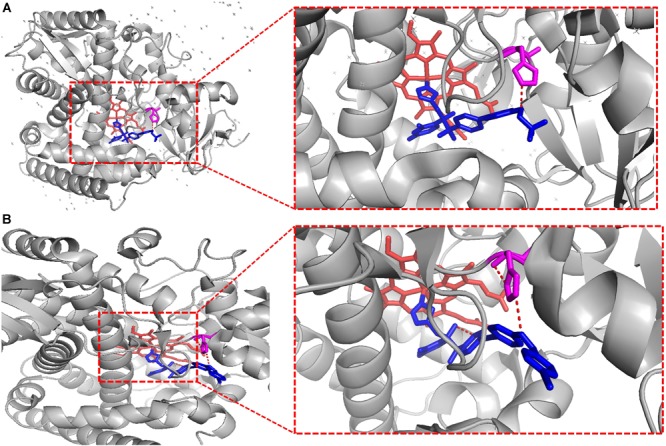
VT-1161 andVT-1598 forming the H bond. VT-1161 and VT-1598 forming the H bond with the His377 of *C. albicans* CYP51 and the His374 of *A. fumigates* CYP51B. **(A)** *C. albicans* CYP51-His377-VT-1161(5TZ1); **(B)** *A. fumigates* CYP51B-His374-VT-1598 (5FRB). They are obtained from the published crystal data file from the PDB database, and then displayed by PyMOL Version 1.5.0.3. The red dot line means H-bonds formed between VT-1161 and *C. albicans* CYP51-His377 in Figure 5A, and H-bonds formed between VT-1598 and *A. fumigates* CYP51B-His374 in Figure 5B.

To crystallize *C. albicans* CYP51 proteins complexes with posaconazole and a tetrazole-based drug candidate VT-1161, Hargrove et al. used *E. coli* cells to express and purify the truncated *C. albicans* CYP51 proteins (56 kDa) without the 48-amino-acid-long N-terminal membrane anchor sequence (Hargrove et al., 2017a). The structural analysis of *C. albicans* CYP51 proteins complexes carried out by Hargrove et al. showed that posaconazole had contacts with a set of 28 residues of *C. albicans* CYP51 proteins, while VT-1161 interacts with 22 amino acid residues, and forms the H-bond between its trifluoroethoxyphenyl oxygen and the imidazole ring of His377 of *C. albicans* CYP51 proteins [Table 2 from Hargrove et al. (2017a) with slight modification]. Further, the X-ray structure of *A. fumigates* CYP51B complex with the tetrazole-based inhibitor VT-1598 (5FRB) determined also by Hargrove et al. (2017b) showed the formation of an optimized hydrogen bond between the phenoxymethyl oxygen of VT-1598 and the imidazole ring nitrogen of His374 of *A. fumigates* CYP51B. Comparative structural analysis of the CYP51 proteins residue (His377 of *C. albicans* CYP51 proteins, His374 of *A. fumigates* CYP51B) among different organisms suggested it was highly conserved across fungal pathogens but not in human, supporting its fungus specificity and the role of H bonding in fungal CYP51/inhibitor complexes (Figure 2, 5).

**Table 2:** Posaconazole and a tetrazole-based drug candidate VT-1161 contacting residues (<4.5 Å) in *C. albicans* CYP51 structures.

	Drug	
Secondary structural element	Posaconazole	VT-1161
	*C. albicans* PDB code 5FSA	C. albicans PDB code 5TZ1
Helix A′	Phe-58	
	Ala-61	
	Ala-62	
	Tyr-64	Tyr-64
	Gly-65	
β1-β2 turn	Leu-88	
Helix B′	Tyr-118	Tyr-118
	Leu-121	Leu-121
	Thr-122	Thr-122
	Phe-126	Phe-126
B″ helical turn	Ile-131	Ile-131
	Tyr-132	Tyr-132
Helix C		
Helix F″	Phe-228	Phe-228
	Pro-230	Pro-230
		
		
	Phe-233	Phe-233
Helix I	Gly-303	Gly-303
	Ile-304	Ile-304
		
	Gly-307	Gly-307
	Gly-308	Gly-308
	Thr-311	Thr-311
K/β1–4 loop	Leu-376	Leu-376
	His-377	**His-377, H-bond**
	Ser-378	Ser-378
β1–4 strand	Phe-380	Phe-380
β4 hairpin	Tyr-505	Tyr-505
	Ser-506	
	Ser-507	Ser-507
	Met-508	Met-508

*Bold is to highlight the hydrogen bond formed between VT-1161 and *C. albicans* CYP51-His377.*

After the first structure of CYP51 proteins with the N-terminal region from *S. cerevisiae* (5EQB) in complex with itraconazole was elucidated and deposited in PDB, Keniya et al. concluded that the conformations of the three full-length fungal Cyp51 structures binding to itraconazole were close to that of the N-truncated *C. albicans* Cyp51 with posaconazole. In comparison these had only slight variations in residues conformations, such as *C. glabrata* Cyp51 I71, T75, I240; *C. albicans* Cyp51 A62, Q66, I231 and *S. cerevisiae* Cyp51 V70, M74, I239, all of which were within the ligand binding pocket and may not be significant due to modest electron densities in this region. Besides, the structures showed *S. cerevisiae* Cyp51-fluconazole or itraconazole had the binding sites in near identical conformations (Sagatova et al., 2015; Keniya et al., 2018). The X-ray crystal structures determined by Keniya et al. (2018) gives us insights into the CYP51 proteins reaction mechanism and emphasizes the identity of ligand-binding sites of fungal CYP51 families, providing a practical basis for the discovery and optimization of novel antifungals targeting at CYP51 families.

## CYP51-Related Drug Resistance

In recent years, with the widespread use of CYP51-targeting drugs, drug-resistant *Candida, Cryptococcus*, and *Aspergillus* have emerged continuously. As shown in Table 3, in many resistant isolates, the decrease of susceptibility originated from mechanisms associated with CYP51, while other isolates not. Besides, transcription factors Pdr1 and Stb5, or the insertion of the Aft1 transposon into the *CYP51* promoter region, can also cause a decrease in the sensitivity of the drugs (Albarrag et al., 2011; Noble et al., 2013; Nishikawa et al., 2016). Transcription factors of non-pathogenic species may also bring some information, such as Set4 in *S. cerevisiae*, which represses *CYP51* expression and reduces drug resistance (Serratore et al., 2018). It is worth noting that drug resistance is often a combination of multiple mechanisms (Berkow and Lockhart, 2017).

**Table 3:** CYP51-related and CYP51-unrelated drug resistance.

Resistance type	Mechanism	Gene(s) involved	Transcription factor(s) involved	Species	References
CYP51 Related	Drug-target point mutation	*CYP51*		*C. albicans; C. tropicalis; C. krusei; C. glabrata; C. auris; C. parapsilosis; C. neoformans; C. gatti; A. fumigatus; A. flavus; S. apiospermum; T. asahii*	1
	Regulation of drug target		Upc2; SREBPs	*C. albicans; C. neoformans; A. fumigatus*	2
	Genomic plasticity	*CYP51*	Upc2;	*C. albicans; C. glabrata; C. neoformans*	3
	Promoter Tandem Repeats	*CYP51*		*A. fumigatus*	4
CYP51 Unrelated	Efflux pump	*CDR1;CDR2;MDR1*	Tac1;Mrr1	*C. albicans; C. glabrata; C. krusei; C. neoformans; A. fumigatus*	5
	Compensatory ergosterol biosynthesis	*ERG3*	Upc2	*C. albicans; C. tropicalis; C. parapsilosis*	6
	Genomic plasticity		Tac1	*C. albicans*	7
	Biofilm formation			*C. albicans; C. glabrata; C. parapsilosis; C. dubiliensis; C. tropicalis; C. neoformans; T. asahii; A. fumigatus*	8
	Activation of Stress Response Passway	*HSP90;PKC1; Calcineurin;TOR*		*C. albicans; C. glabrata; C. neoformans A. terreus A. terreus; A. fumigatus; Paecilomyces variotii; Mucor* spp.	9

*1: Albarrag et al., 2011; Becher and Wirsel, 2012; Gast et al., 2013; Silva et al., 2016; Kushima et al., 2017; Lockhart et al., 2017; Bernhardt et al., 2018; Sharma et al., 2018. 2: Willger et al., 2008; Chang et al., 2009; Flowers et al., 2012; Whaley et al., 2014; Jiang et al., 2016; Pais et al., 2016. 3: Kwon-Chung and Chang, 2012; Parker et al., 2014. 4: Spiess et al., 2014; Gsaller et al., 2016. 5: Cannon et al., 2009. 6: Martel et al., 2010; Eddouzi et al., 2013; Branco et al., 2017. 7: Morio et al., 2017. 8: Desai et al., 2014. 9: Wong et al., 1998; Cruz et al., 2001; Blankenship et al., 2003; Cowen et al., 2009; Singh-Babak et al., 2012; Lamoth et al., 2015.*

The mechanisms associated with CYP51 are discussed below in detail.

### *Candida* spp.

*Candida* spp., represented by *C. albicans*, is the most prominent pathogenic fungus. The spectrum of disease of invasive candidiasis ranges from minimally symptomatic candidaemia to fulminant sepsis with an associated mortality exceeding 70% (Pappas et al., 2018). The resistance mechanisms of *Candida spp.* related with CYP51 includes point mutation, genomic plasticity, and upregulation of *CYP51* meditated by the transportation factor Upc2.

Point mutation (amino acid non-synonymous substitutions) of CYP51 proteins is a critical origination of reduced drug susceptibility. Three ways were proposed to explain the resistance mechanism: (1) the corresponding amino acids docked with azoles are changed, (2) the structure of the binding cavity is rearranged, leading to changed position of the azole molecule or the heme iron, disturbing the interaction between them, and (3) the access of the drug into the active site is blocked (Becher and Wirsel, 2012).

In *C. albicans*, mutations of CYP51 proteins amino acid sequences frequently occurs on 105–165, 266–287, and 405–488 (Marichal et al., 1999). Among these mutations, N-terminal and C-terminal are more frequent than central regions, probably due to these terminal substitutions lead to changes in secondary and tertiary structure, especially substrate binding cavities, which are located in α-helix B, B′, C, and in their connection loops (Becher and Wirsel, 2012). And sequential replacement of ERG11 mutant alleles with wild-type alleles contributes to the reduction of resistance (MacCallum et al., 2010). The resistant *C. albicans* double point mutation Y132F G464S (Y140F G464S by *S. cerevisiae* numbering) were artificially introduced into *S. cerevisiae* CYP51, leading to a decrease in sensitivity of the latter (Sagatova et al., 2018). In addition, mutations located at different sites of CYP51 proteins provide different degrees of resistance enhancement. As an instance, K143R is stronger than F449V (Flowers et al., 2015).

The non-albicans *Candida* (NCAC) species also contains amino acid non-synonymous substitutions, such as Y132F in *C. parapsilosis*, Y132F and K143R in *C. tropicalis* (Vandeputte et al., 2005; Xisto et al., 2017; Choi et al., 2018).

The newly emerged *C. auris* is a multi-drug resistant *Candida* that causes serious invasive infections, with a mortality rate approximate to 60% (Lee et al., 2011). *CYP51* point mutation plays an important role in pan-azole resistance of *C. auris*. Nine typical amino acid substitutions have been identified, three of which are closely associated with geographic clades: F126T in South Africa, Y132F in Venezuela, and Y132F or K143R in India and Pakistan (Lockhart et al., 2017).

Genomic plasticity, including aneuploidy and loss of heterozygosity (LOH), refers to the abnormal fungal chromosomal behavior induced by heat, oxidative stress or antifungal drugs. It is an important mechanism for fungal adaptation to the environment.

Aneuploidy is a reversible fungal chromosomal adaptive behavior in response to drug stress. If the “selectivity” of the drug disappears, the cells will return to the euploid state. Aneuploidy after azole stress was first discovered in *C. glabrata* and was detailed studied in *C. albicans* (Vanden et al., 1992).

The aneuploidy of *C. albicans* chr1, chr3, chr5, chr6 and chrR has been reported under the stress of azoles treatments (Selmecki et al., 2010; Li et al., 2015). Research on fluconazole-resistant strains has revealed that at least half of them carry aneuploid chromosomes. The duplication of the chr5’s left arm in *C. albicans* [named i(5L)] results in multiple copies and overexpression of *CYP51* gene, contributing to azole resistance (Selmecki et al., 2006). Besides, aneuploidy of chr5 (carrying *TAC1*), chr3 (*MRR1* and *CDR1*), Chr4 or chr6 (*MDR1*) also reduce drug susceptibility (Selmecki et al., 2010). Some aneuploid *C. albicans* chromosomes source from the mitotic defect in the process of quasi-fertility (Forche et al., 2008). There also exists an additional mechanism that, two diploid cells and one tetraploid cell are connected to each other to form a “trimeric,” then the tetraploid cell undergoes defective mitosis and results in two aneuploid progeny cells (Harrison et al., 2014).

An “Evolution Trap” (ET) strategy was proposed to suppress the occurrence of aneuploidy. The aneuploidy of a whole microflora can develop into multiple random directions, but once a specific inducing factor (stress X) is used to strictly limit its development direction, another treatment (treatment Y) can be applied to eliminate this trend and inhibits the generation of resistance. Such strategy has successfully pulled the minimum inhibitory concentration (MIC) against *C. albicans* carrying aneuploidy-sourced resistance back to normal level (Chen et al., 2015).

Loss of heterozygosity is another branch of genomic plasticity leading to *Candida* species resistance. It is an irreversible process in diploid fungi, thus resulting the acquired resistance gene mutation (^∗^) become multiple (e.g., *ERG11/ERG11^∗^→ ERG11^∗^/ ERG11^∗^*), thus results in overexpression of such gene (White, 1997). LOH contains three mechanisms, (1) local recombination of chromosomes, (2) mitotic recombination between centromeres and related locus, (3) whole-chromosome loss and the remaining chromosome’s duplication (Morschhäuser, 2016).

Transcription factors of *CYP51* are tightly related to drug resistance. The Zn_2_-Cys_6_ transcription factor Upc2, located in *Candida* spp., is highly relevant to the increase of azole sensitivity (Vasicek et al., 2014). It has been confirmed that when Upc2 in *C. albicans* or *C. parapsilosis* or Upc2A in *C. glabrata* is deleted, the susceptibility to azoles will be enhanced (Guida et al., 2011; Vasicek et al., 2014; Whaley et al., 2014). The C-terminal domain (CTD) of Upc2 is a novel α-helical fold with a deep hydrophobic pocket. Treatment with azole reduces the membrane ergosterol level, then ergosterol molecules that are previously bound to CTD dissociates from Upc2p. Thereby Upc2p relocates from the cytoplasm to the nucleus to activate *CYP51* expression (Yang et al., 2015). It is worth noting that Upc2 only up-regulates the expression of *CYP51* under azole stress conditions (Hoot et al., 2011).

The gain-of-function (GOF) mutation of Upc2 also contributes to *Candida* species increased drug resistance. Typical GOF point mutations inducing overexpression of *CYP51* in *C. albicans* include A643V, G648D, G648S, and Y642F (Dunkel et al., 2008; Flowers et al., 2012). Point mutations in UPC2 can reduce the sensitivity to azoles in combination with amino acid substitution of CYP51. For example, when combined with the CYP51^G464S^ mutation, the MIC value of *C. albicans* carrying Upc2^G648D^ against fluconazole is increased from 4 μg/ml to 16 μg/ml (Sasse et al., 2012).

Gain-of-function mutations were also found in *C. tropicalis* Upc2. Nucleotide substitutions T118G and G155A in *CtUpc2* promoter increase the expression of this gene, and amino acid substitution G392E in CtUpc2p enhances drug resistance when expressed heterologously in *S. cerevisiae* (Jiang et al., 2016).

The GOF amino acid substitutions are often localized near the C terminus of Upc2p, where the activation domain of zinc-cluster transcription factors is found. Mutations in this region leading to reduced drug susceptibility include two possible mechanisms: (1) relieve Upc2 from a repressor that would otherwise keep this transcription factor inactive, (2) interfere with the transmembrane region of this protein, leading to Upc2p nuclear localization and initiation of *CYP51* transcription (Flowers et al., 2012).

### *Cryptococcus* spp.

Cryptococcal meningitis (CM) is the most common infection caused by *Cryptococcus* spp., and is frequently recommended to be treated with fluconazole monotherapy in national guidelines. However, even with such treatment, the mortality of CM can exceed 50%, much of which owing to drug-induced *cryptococcal* resistance (Rothe et al., 2013; Bongomin et al., 2018). Amino acid point mutation, genomic aneuploidy and Sre1-induced overexpression of *CYP51* account for the reduced azole susceptibility.

The amino acid point mutation G344S was found in CYP51 proteins of *Cryptococcus neoformans var. grubii*, resulting in multi-azole resistance (Kano et al., 2017). Besides, the substitution G484S may confer a change in the orientation of the P450 heme-binding domain, decreasing catalytic activity and azole binding of *Cryptococcus neoformans* CYP51 proteins (*Cn*CYP51 proteins) (Rodero et al., 2003). And the substitution Y145F affords resistance to voriconazole but attenuates resistance to itraconazole and posaconazole (Sionov et al., 2012). In *Cryptococcus gattii* CYP51 proteins, amino acid non-synonymous substitution N249D are deduced to result in azole resistance (Gast et al., 2013).

As for genomic aneuploidy, *Cryptococcus neoformans* Chr1 disomy, which is common in heteroresistant isolates, results in duplication of *CnCYP51* and reduced azole sensitivity. A recent report reveals that clinical fluconazole treatment can induce aneuploidy of *C. neoformans* in CM patients, and relapse of CM is associated with Chr1 disomy (Bongomin et al., 2018; Stone et al., 2019).

*Cryptococcus neoformans* is haploid and aneuploid cells of this species mainly derive from the uncoupling of cell growth and nuclear division (Altamirano et al., 2017). Unlike *C. albicans*, the aneuploidy of Chr1 in *C. neoformans* only repeats the entire chromosome without forming segmental isochromosomes (Kwon-Chung and Chang, 2012). A study indicates the detailed mechanism causing aneuploidy: final degradation of the septum is affected by fluconazole during cytokinesis, resulting in Chr1 disomy multinucleated cells, and these cells exhibit an increased potential to proliferate in the presence of fluconazole (Altamirano et al., 2017). However, another study points out that the fluconazole-induced multinucleated cells fail to propagate to form colonies in the presence of fluconazole, and chromosome missegregation of *C. neoformans* dividing cells has not been detected, suggesting *C. neoformans* forms aneuploid clones directly from uninucleated cells under fluconazole stress (Chang et al., 2018).

Aneuploidy can be regulated by certain factors in *Cryptococcal* spp. For example, the decreased expression of *AIF1* (apoptosis-inducing factor) conduces to maintain Chr1 aneuploidy, thereby contributes to a stable repeat of *CYP51* and preserves resistance to azoles (Semighini et al., 2011).

Sterol regulatory element-binding protein (SREBPs) regulate the *CYP51*s’ expression in many species of fungal, including *C. neoformans* (Chang et al., 2009). Under azoles or hypoxic stress, SREBPs regulate the transcription of *CYP51* by binding to the sterol regulatory element (SRE) in the promoter

The SREBP in *C. neoformans* is Sre1. Sre1 is cleaved by Scp1and functions to regulate the expression of *CYP51*, thus plays a key role in drug resistance (Bien and Espenshade, 2010). Studies have shown that deletion of Sre1 converts the effect of azoles from fungistatic to fungicidal (Bien et al., 2009; Chang et al., 2009).

### *Aspergillus* spp.

The ability *Aspergillus* spp. to adapt to mammal hosts or external environment is a vital fungal characteristic that leads to treatment failure and the emergence of resistant isolates worldwide. Non-synonymous substitution of amino acids, transcription factors SrbA and AtrR, tandem repeats, and Dap proteins constitute the CYP51-related resistance mechanisms of *Aspergillus* spp.

The lanosterol 14α-demethylase point amino acid mutations of *A. fumigatus* mainly appear in CYP51A, and G54, L98, G138, M220 and G448 are the hotspots (Denning and Perlin, 2011). G54R/E/V and G138 lead to cross-resistance to itraconazole and posaconazole, and G448S results in voriconazole tolerance (Chowdhary et al., 2017), while M220I/V/T/K can develop resistance to itraconazole, voriconazole, refconazole, and posaconazole (Mellado et al., 2004). When the G138S point-substituted *CYP51A* in the resistant *A. fumigatus* was mutated back, the tolerance of the isolate diminished (Umeyama et al., 2018). Moreover, one mutation may have varied effects on resistance for different azoles. For example, in the heterologous expression experiment, G54W significantly reduces the susceptibility to itraconazole and posaconazole, while has almost no effect on voriconazole (Alcazar-Fuoli et al., 2011).

Amino acids substitution also occurs in non-fumigatus *Aspergillus* species. Four mutations of *A. flavus* CYP51C (S196F, A324P, N423D and V465M) are correlated with voriconazole resistance (Sharma et al., 2018). For *A. clavatus* CYP51A, E483K and P486S mutations may narrow the azole transport and therefore confer lower susceptibility (Abastabar et al., 2019). As for *A. terreus* and related species, M217T and M217V mutations of CYP51Ap was found correlating with posaconazole resistance (Zoran et al., 2018).

Notably, appliance of azole pesticides in agriculture is one of the reasons for the non-synonymous substitution of *A. fumigatus* CYP51 proteins. Mutations in this type include L98H, Y121F and T289A, which are often accompanied with tandem repeats of the *CYP51* promoter (Mellado et al., 2007; Camps et al., 2012; Chowdhary et al., 2013; van der Linden et al., 2013; Isla et al., 2018).

Tandem repeats include 34-base pair (TR34) and 46-base pair (TR46) (Spiess et al., 2014). CBC (CGAAT binding complex) binds to CGAAT of -293 to -289 position in the CYP51 promoter and downregulates *CYP51A* expression. Tandem repeats reduce the affinity of CBC and the promoter, upregulating *CYP51A*. Mechanism researches indicate that the presence of eight different nucleotides at the 3′end of TR34 lead to lower CBC affinity (Gsaller et al., 2016).

The combination of *CYP51* promoter tandem repeat and CYP51 proteins point mutation contains TR34/L98H/S297T/F495I and TR46/Y121F/T289A, leading to broad-spectrum azole resistance. (Snelders et al., 2015; Chen et al., 2018; Isla et al., 2018; Pinto et al., 2018; Tsitsopoulou et al., 2018; Tsuchido et al., 2019). Studies on TR34/L98H have shown that L98H can cause a flexible change in the BC loop and IH loop of *A. fumigatus* CYP51A (*Af*CYP51A), which changes the position of the tyrosine107 and tyrosine 121 side chains. This modifies the ligand access channels in the *Af*CYP51A and prevents the binding of azoles toward the active heme (Snelders et al., 2011). Moreover, TR34/L98H doesn’t incur a fitness cost or survival disadvantage to *A. fumigatus* (Beer et al., 2018). On the other hand, studies on TR46/Y121F/T289A indicate that the Y121F substitution seems to disrupt the H-bond between tyrosine and the heme center of *Af*CYP51A, resulting in the instability of enzyme’s active center (Snelders et al., 2015).

Besides, the insertion of Atf1 in the *A. fumigatus CYP51A* promoter may also be one of the factors leading to azole resistance (Albarrag et al., 2011). But it requires more researches to confirm whether the effect is direct.

The transportation factor SrbA, as one of *A. fumigatus* SREBPs, modulates the expression of *AfCYP51A*. Different from *C. neoformans*, Scap homologue is absent in *A. fumigatus*, and SrbA directly binds to the 34 mer of *AfCYP51A* promoter without cleavage, regulating the synthesis of ergosterol (Blosser and Cramer, 2011; Gsaller et al., 2016). SrbA is directly activated by azole stress and is associated with the intrinsic resistance of *A. fumigatus* to fluconazole (Song et al., 2017). An azole-resistant strain with TR46/Y121F/T289A can be sensitized to azoles by deletion of srbA (Hagiwara et al., 2016). The intrinsic expression of *CYP51* in such strain restores the MIC value to its original level (Willger et al., 2008).

AtrR is a newly discovered fungal-specific Zn2-Cys6 transcription factor in *Aspergillus* spp. It modulates the expression of *CYP51A* by directly binding to the promoter of this gene. Deletion of AtrR results in hypersensitivity to azoles and invalidates the CYP51A^G54E^ mutation that would otherwise render azole resistance (Hagiwara et al., 2017).

Damage Resistance Protein A (DapA), which belongs to the cytochrome b5-like heme-binding damage resistance protein (Dap) family, responds to azole treatment in a concentration-dependent manner in *A. fumigatus*. It co-localizes with CYP51A/B protein in the endoplasmic reticulum (ER), and then binds to the heme group to stabilize these CYP51 proteins. DapA is highly associated with the intrinsic azole resistance of *A. fumigatus*, and the deletion of *DapA* leads to hypersensitivity to azoles (Song et al., 2016). Studies show that DapA may indirectly sense the azole stress at the downstream of SrbA (Song et al., 2017).

## Traditional and Novel CYP51-Targeting Antifungal Agents

The study of azole antifungal antifungal agents can be traced back to 1944 (Woolley, 1944). Since then, imidazole (clotrimazole, miconazole, econazole, and ketoconazole) and two generations of triazoles (fluconazole, itraconazole, voriconazole, posaconazole, isavuconazole) successively entered into the clinic (Musiol and Kowalczyk, 2012) (partly shown in Table 4). Besides, there are several other CYP51 inhibitors used in the treatment of topical or superficial fungal infections, such as oxiconazole, sertaconazole, luliconazole, efinaconazole, and ravaconazole.

**Table 4:** Current antifungal CYP51 inhibitors.

Name	Approval time	Clinical applications	Adverse effects	Remarks	References
Ketoconazole(KCZ)	1981	Oral, vaginal, cutaneous and systemic candidiasis	Liver damage, interference of the endocrine system, nausea, headache, abdominal pain, etc.	Oral formulation has withdrawn from Europe, Australia, and China.	10
Fluconazole(FCZ)	1988	Systemic *Candida* infection, cryptococcal meningitis, vaginal, oropharyngeal and esophageal candidiasis	Nausea, vomiting, abdominal pain, gastrointestinal adverse reactions, reversible mild liver necrosis and thrombocytopenia	Adjuvants such as calcineurin inhibitors, heat shock protein 90 inhibitors have been found	11
Itraconazole(ICZ)	1988	Invasive aspergillosis, superficial candidiasis, dermatophyte infection, sporotrichosis, blastomycosis, histoplasmosis, penicilliosis, and coccidioidomycosis, etc.	Gastrointestinal symptoms, cardiac failure, peripheral edema and hepatic inflammation		12
Voriconazole(VCZ)	2002	Aspergillosis, candidiasis, scedosporium, and *Fusarium* infection	Neurotoxicity, visual toxicity, hepatotoxicity and skin malignancy	Therapeutic drug testing (TDM) is required	13
Posaconazole(PCZ)	2006	*Aspergillus* and *Candida* infections, especially fluconazole or itraconazole resistant cases	Serious adverse reactions rarely exit. Non-serious adverse reactions include SeHeadache, nausea, and menstrual disorder, etc.		14
Isavuconazole	2015	Invasive aspergillosis and invasive mucormycosis	Nausea, vomiting, diarrhea and hepatobiliary toxicity		15

*10: Listed, 1981; Heel et al., 1982; Pont et al., 1982, 1985; Daneshmend and Warnock, 1988; Chien et al., 1997; Rodriguez and Acosta, 1997; Rodriguez et al., 1999; Greenblatt et al., 2011; Yan et al., 2013; Greenblatt and Greenblatt, 2014; Gupta et al., 2014; Gupta and Lyons, 2015). 11: Washton, 1989; Morita et al., 1992; Amichai and Grunwald, 1998; Fischer et al., 2010; Liu et al., 2014; Behtash et al., 2017. 12: Isoherranen, 2004; Lestner and Hope, 2013; Abuhelwa et al., 2015. 13: Hyland et al., 2003; Wood et al., 2003; Espinel-Ingroff et al., 2012; Owusu et al., 2014; Malani et al., 2015; Job et al., 2016; Lamoureux et al., 2016; Levine and Chandrasekar, 2016; Patterson et al., 2016; Li et al., 2017; Xing et al., 2017; Cormican et al., 2018; Mounier et al., 2018. 14: Kim et al., 2003; Sansone-Parsons et al., 2007; Groll and Walsh, 2014; Clark et al., 2015; Moore et al., 2015. 15: Schmitt-Hoffmann et al., 2006; McCormack, 2015; Pettit and Carver, 2015; Traynor, 2015; Maertens et al., 2016; Denis et al., 2018; Ledoux et al., 2018.*

**FIGURE 6:**
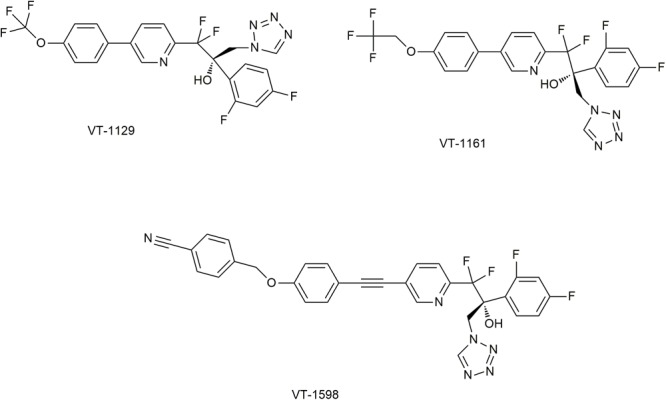
Novel CYP51 inhibitors in studies. It has been permitted by the copyright holders through RightsLink.

Both imidazoles and triazoles exits adverse reactions caused by inhibition of human CYP450 (such as CYP3A4 and CYP2C9), due to the strong affinity to heme iron (Hoekstra et al., 2014). Fortunately, replacement by 1-tetrazole could attenuate such affinity. Based on this idea, new compounds VT-1611, VT-1129, and VT-1598 have been developed (Hoekstra et al., 2014) (Figure 6).

### VT-1598

VT-1598 has a good affinity for the fungal CYP51 proteins, as the K_d_ of this compound to *A. fumigatus* CYP51B is 13 nM (Hargrove et al., 2017b). Its antifungal spectrum is relatively broad. The MIC_50_ for azole-resistant *C. albicans* and *C. glabrata* is 0.124 and 1.19 μg/ml (Wiederhold et al., 2018b). Especially, the inhibition of VT-1598 against the clinical *Coccidioides* isolates is significantly better than that of fluconazole (McCarthy et al., 2017). The non-selective inhibition of VT-1598 to human CYP450 is weak, as IC_50_ for human CYP2C9 and CYP3A4 are both more than 200 μM, and the IC_50_ for CYP2C19 is 138 μM (Yates et al., 2017). When the oral dose (15 mg/kg/day) of VT-1598 is applied to mice, the C_max_ is about 13 mg/L, the C_24__h_ level is 6.7 mg/L and the half-life period is 22 h in plasma (Garvey et al., 2018).

VT-1598 has a high binding rate of plasma protein. When co-incubated with plasma at concentration of 1 mg or 5 mg/L *in vitro*, only less than 1% showed a free state (Garvey et al., 2018). *In vivo*, when treated with 20 mg/kg of VT-1598 per day, the minimum plasma concentration (C_min_) was 32-fold greater than that of the 25 mg/kg dose of fluconazole (Break et al., 2018a).

In the mouse model, VT-1598 has been used alone or in combination with amphotericin liposomes, and has achieved good efficiency for cryptococcal meningitis caused by *C. neoformans* or *C. gatti* infection (Garvey et al., 2018). VT-1598 has also achieved significant preventive effects against mucosal candidiasis induced by sensitive or resistant *Candida* spp. (Break et al., 2018a). In addition, VT-1598 can be used to treat central nervous system coccidioidomycosis infected by *C. posadasii* and *C. immitis* (Wiederhold et al., 2018c). In May 2016, FDA granted orphan drug designation to VT-1598 for the treatment of Valley fever, a disease caused by *Coccidioides* infection (McCarthy et al., 2017).

### VT-1161 (Oteseconazole)

VT-1161 can tightly bind to the fungal CYP51 proteins and effectively inhibit the activity of such proteins. For example, the K_d_ of VT-1161 to *C. albicans* CYP51 proteins is less than 39 nM and IC_50_ ranges from 1.4 to 1.6 μM, resulting in the proportion of ergosterol to total sterols drop to only 3% (Warrilow et al., 2014). The MIC_50_ of VT-1161 against fluconazole-resistant *C. albicans* is 0.03 μg/ml (Break et al., 2018b). For *T. rubrum* CYP51 proteins, The K_d_ and IC_50_ values are 242 nM and 0.14 μM, respectively (Warrilow et al., 2017). Its non-selective inhibition of human CYP450 is also weak, appears in its IC_50_ of CYP2C9, CYP2C19 and CYP3A4 are 99, 72, and 65 μM. Pharmacokinetic experiments in mice showed that VT1161 has a wide distribution volume (1.4 L/kg), high oral bioavailability (73%), and long half-life period (>48 h)(Garvey et al., 2015). Phase II clinical trials showed oral VT-1161 plasma exposure for the 150 mg/24 weeks’ or 300 mg/24 weeks’ treatment groups were 3.81 and 8.33 μg/mL (Brand et al., 2018).

Animal model studies have shown that VT1161 can be used to prevent or treat mucormycosis caused by *Rhizopus arrhizus*, and can also be used for treatment of mouse modeling infection or canine naturally occurring coccidioidomycosis (Shubitz et al., 2015, 2017; Gebremariam et al., 2017). In addition, in a mouse model, VT1611 can treat oropharynx or vaginal Candidiasis caused by fluconazole sensitive or resistant *C. albicans* (Break et al., 2018b). Phase II clinical trials indicate that oral VT1161 has a good effect on treating human vulvar candidiasis, with a satisfactory tolerance and a low incidence of adverse reactions (Brand et al., 2018). Currently, oral VT-1161 has completed Phase II clinical trials for moderate – severe interdigital tinea pedis, vaginal candidiasis, and onychomycosis of the toenail. And phase III clinical trial is underway for Vaginal Candidiasis.

### VT-1129 (Quilseconazole)

VT-1129 has a similar skeleton to VT-1161, and shows a remarkable inhibitory activity against *Cryptococcus* CYP51 protein. For example, the Kd for *C. neoformans, C. gattii*, and *C. grubii* CYP51 proteins are about 11, 24, and 25 nM, respectively, and the corresponding IC50 are 0.16, 0.15, and 0.18 μM. VT-1129 can reduce the proportion of ergosterol to total sterols in C. neoformans to 11.5%, and only 0.12 μg/ml can totally inhibit the growth of *C. neoformans* (Warrilow et al., 2016; Wiederhold et al., 2018a). Its non-specific inhibition to human CYP450 is lower than that of the previous azole antifungals, appears in the IC50 to CYP2C9 and CYP2C19 are 87 and 110 μM, respectively, and for CYP3A4 is also higher than 79 μM (Warrilow et al., 2016). VT-1129 also inhibits the growth of azole or echinocandin resistant *C. glabrata* and *C. krusei* (Schell et al., 2017).

The pharmacokinetic experiments in mice model showed that VT-1129’s half-life period is long (>6 days). The plasma and brain concentrations were still above the MIC values even after 20 and 32 days stopping oral treatment of VT-1129. The non-linear pharmacokinetic model has been approved to describe the correlation between concentrations of VT-1129 in plasma and in brain (Wiederhold et al., 2018a,d).

At present, the research on VT-1129 in animal models is mainly focused on cryptococcal meningitis. VT-1129 significantly reduces fungal burden and improve survival rates during treatments. When treated with a dose of ≥ 3 mg/kg/day, the fungal burden was undetectable in most mice even 20 days after dosing was stopped. And treating at a dose of 20 mg/kg once daily reached a maximal survival benefit (100%). Because VT-1129 plasma and brain concentrations are related with fungal burden reductions, the loading dose-maintenance dose (LD-MD) strategy to treat cryptococcal meningitis seems feasible (Wiederhold et al., 2018a,d).

## Conclusion and Perspectives

CYP51 plays a crucial role in fungal invasive growth, hyphae formation and virulence, and inhibitors targeting CYP51 have always been an important component of antifungal agents. Further researches on fungal CYP51s might set about from the following aspects: First, while the detailed crystal structures of several susceptible pathogenic fungi CYP51 have been elaborated, those of drug-resistant variants are still in hypothesis. If those structures were elucidated, targeting at common drug-resistant CYP51 protein variants could probably be promising. Second, it deserves more in-depth researches to find out why some kind of amino acid point mutations (such as G54W in *A. fumigatus CYP51A*) could have varied effects on resistance for different azoles. Such researches may provide important ideas to minimize drug resistance. Third, the study of model organisms may also bring some inspiration. Such as Set4, which targets to ergosterol gene promoters with a Hap1-dependent manner under hypoxic conditions in *S. cerevisiae*, could downregulates the expression of *CYP51*. Whether homologous proteins and similar mechanisms exist in pathogenic fungi requires further researches. As long as they exist, new remedies may be put forward to activate Set4 to inhibit azole-induced *CYP51* overexpression. Forth, it might be achievable to target other proteins as well as CYP51 simultaneously to maximize the therapeutic effect. There already exists some preliminary works focusing on this, and it is probably requisite to find out more accompanying targets. Fifth, the ”Evolution Trap”(ET) strategy, which successfully restored the sensitivity of *C. albicans in vitro*, might be practiced *in vivo* to investigate whether similar effects exist in animal models and further in humans. Aside from these, it is also hopeful for pharmaceutics amelioration to improve treatment efficacy. Novel drug delivery systems, such as liposomes, may confer better oral bioavailability on azoles.

## Author Contributions

JZ contributed to the section of CYP51’s functions, inhibitors and drug resistance. LL contributed the structural analysis part of CYP51. QL, LY, YW, and YJ provided the revisions for this article.

## Conflict of Interest Statement

The authors declare that the research was conducted in the absence of any commercial or financial relationships that could be construed as a potential conflict of interest.
